# The effect of silica desiccation under different storage conditions on filter-immobilized environmental DNA

**DOI:** 10.1186/s13104-021-05530-x

**Published:** 2021-03-21

**Authors:** Michael J. Allison, Jessica M. Round, Lauren C. Bergman, Ali Mirabzadeh, Heather Allen, Aron Weir, Caren C. Helbing

**Affiliations:** 1 Department of Biochemistry and Microbiology, University of Victoria, Victoria, BC Canada; 2 Bureau Veritas Laboratories, Guelph, ON Canada; 3 Bureau Veritas Laboratories, Burnaby, BC Canada

**Keywords:** Environmental DNA, Filter, Long-term storage, Silica gel beads, Ethanol, Quantitative real time polymerase chain reaction, Storage conditions

## Abstract

**Objective:**

Silica gel beads have promise as a non-toxic, cost-effective, portable method for storing environmental DNA (eDNA) immobilized on filter membranes. Consequently, many ecological surveys are turning to silica bead filter desiccation rather than ethanol preservation. However, no systematic evaluation of silica bead storage conditions or duration past 1 week has been published. The present study evaluates the quality of filter-immobilized eDNA desiccated with silica gel under different storage conditions for over a year using targeted quantitative real-time polymerase chain reaction (qPCR)-based assays.

**Results:**

While the detection of relatively abundant eDNA target was stable over 15 months from either ethanol- or silica gel-preserved filters at − 20 and 4 °C, silica gel out-performed ethanol preservation at 23 °C by preventing a progressive decrease in eDNA sample quality. Silica gel filter desiccation preserved low abundance eDNA equally well up to 1 month regardless of storage temperature (18, 4, or − 20 °C). However only storage at − 20 °C prevented a noticeable decrease in detectability at 5 and 12 months. The results indicate that brief storage of eDNA filters with silica gel beads up to 1 month can be successfully accomplished at a range of temperatures. However, longer-term storage should be at − 20 °C to maximize sample integrity.

**Supplementary Information:**

The online version contains supplementary material available at 10.1186/s13104-021-05530-x.

## Introduction

Over the past decade, techniques used to detect environmental DNA (eDNA)—genetic material present in environmental samples from secretions, excretions, exogenous sloughing of eukaryotic cells, or from microscopic organisms [1]—have surged in use by academic, government, conservation, and development sectors for providing cost-effective information about at-risk and invasive species in natural and managed ecosystems [2–5]. In eDNA-based surveys, a common technique is to immobilize eDNA from water samples on filter membranes, extract the DNA, and perform targeted taxa quantitative real-time polymerase chain reaction (qPCR) analyses. Ideally, these filters can be stored for some time or archived prior to DNA analysis. Filters are typically immersed in high percentage, molecular grade ethanol to prevent sample degradation during storage [6]. Immersion of filters in ethanol at room temperature for up to 2 weeks gave better eDNA performance than directly freezing filters with no preservation or extracting DNA from filters within 5 h of filtration at room temperature [7]. While relatively straightforward to use, the use of ethanol presents several challenges in that it is a dangerous good (volatile, flammable, and poisonous) requiring special shipping permits, and adds considerable bulk and weight to the field sample. However, recently, the use of silica gel beads as a filter desiccant have been suggested as a lightweight alternative to ethanol immersion [8–10]. Despite this suggestion, little empirical work has been published evaluating silica gel filter storage conditions under different temperatures over periods greater than 1 week.

The present study evaluates the quality of filter-immobilized eDNA desiccated with silica gel beads for over a year under different storage temperatures. In the first experiment, targeted detection of relatively abundant (~ 50,000 copies per reaction) eDNA from an outdoor freshwater tank was examined using filters preserved in ethanol or silica gel beads for up to 15 months at 23, 4, and − 20 °C. In the second experiment, low abundance (~ 500 copies per reaction) eDNA targets from water samples spiked with a dilute tissue slurry were tracked using two separate targeted qPCR assays on filters preserved by silica gel beads for up to 12 months at 18, 4, and − 20 °C.

## Main text

### Methods

#### Animal care and handling

A single premetamorphic American bullfrog (*Rana [Lithobates] catesbeiana*) tadpole was used as a tissue source to create a standard slurry detailed below. The tadpole was euthanized using 0.1% (w/v) tricaine methanesulfonate (Syndel Laboratories, Nanaimo, BC, Canada) buffered in 25 nM sodium bicarbonate (Sigma Aldrich, Canada).

#### Filter storage in silica gel beads

All procedures were performed in an amplicon-free area and the benchtop was wiped with 10% bleach (v/v) (Javex 12 by Clorox) solution followed by 70% ethanol (v/v) prior to setup. Personnel wore nitrile gloves, safety glasses, and a lab coat. In all procedures, forceps were submerged in 50% bleach (v/v) and thoroughly rinsed with distilled water and dried between sample handling events. Sample water (details below) was vacuum filtered through Nalgene analytical test filter funnels with 0.45 μm mixed cellulose ester filters (Thermofisher Scientific Inc., Mississauga, ON, Cat#145-2045). Mixed cellulose ester (cellulose nitrate and acetate mixture) was chosen because it, along with cellulose nitrate, empirically gave the highest DNA yield when comparing filter membrane compositions [7, 9, 11, 12]. The vacuum was maintained for 1 min after the sample had passed through to remove excess water. Filters were preserved whole to replicate the effects of disturbance during repeat DNA isolations where one-quarter of a filter is broken off for every round of DNA isolation.

**Fig. 1:**
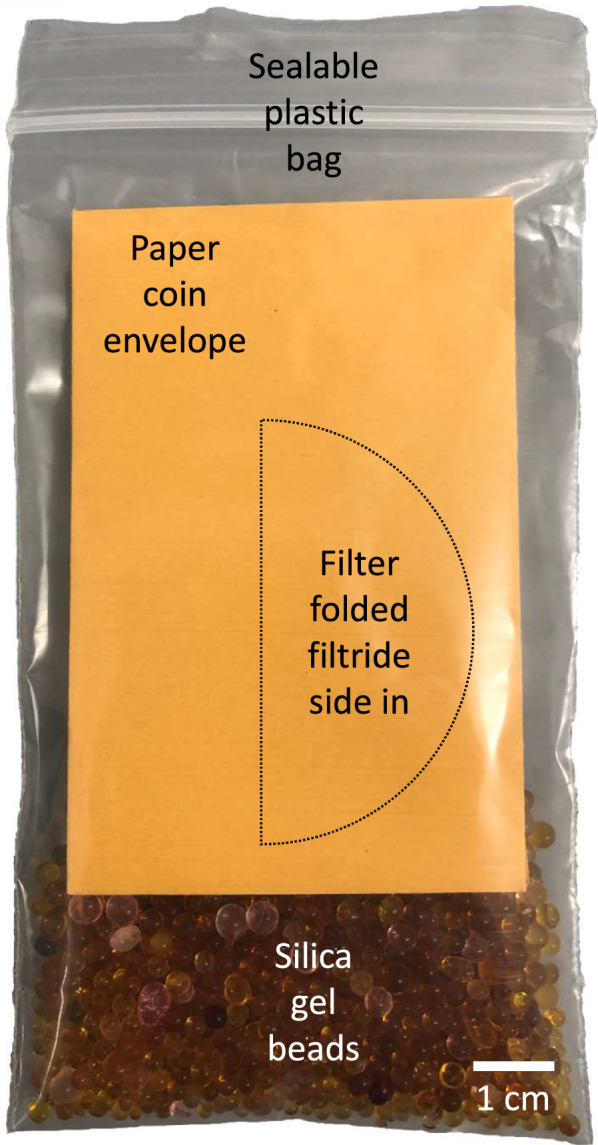
Recommended method for storing an eDNA filter sample using silica gel beads. A filter membrane is folded in half with the filtride side in and placed inside a paper coin envelope. This, in turn, is placed in a sealable plastic bag containing silica gel beads, the air removed and firmly sealed before storage. It is easy to monitor the moisture content of the sample as the orange beads turn dark green when there is too much moisture and need to be replaced or the beads regenerated as per the manufacturer’s instructions

Using forceps, the filter was folded in half with the filtride facing inward and inserted into a pre-labelled Manila paper coin envelope (Fig. 1). This was then inserted into a small sealable plastic bag to which 15–30 mL of color-indicating 2–4 mm rechargeable silica gel beads (Dry & Dry, Amazon, Canada; Product #CRH-16036) were added. The orange beads will turn dark green when they are 50–60% water saturated allowing for easy monitoring of desiccation conditions during storage. The coin envelope prevents direct contact between the filter and the silica beads and protects the filter from damage. The filters, thus prepared, were stored in the dark at the indicated temperatures.

#### Experiment 1: Comparison between ethanol and silica filter preservation of relatively abundant eDNA at three holding temperatures over time

One-litre samples (n = 2–3 per temperature group) of recirculated water were obtained on the same day at the University of Victoria’s Aquatics Facility from an outdoor fiberglass tank used for holding bullfrog tadpoles fed daily with Spirulina. Water was filtered and the filters handled as described above for silica gel desiccation above or folded in quarters and placed into a 2 mL microcentrifuge tube containing 95% molecular-grade ethanol [8]. The tube was filled with ethanol and wrapped in Parafilm for storage to prevent evaporation. One set of filters were not preserved and rather set aside for immediate processing. DNA was extracted from the filters immediately or after 1, 4, 8, or 15 months of storage at − 20, 4, and 23 °C. At each time-point, each filter was individually removed from its coin envelope in a laminar flow hood, and a quarter partitioned off at room temperature. The remaining filter was returned to its coin envelope or the tube containing ethanol and placed back into its designated storage condition.

#### Experiment 2: Effect of holding temperature and time on low abundance eDNA on silica-preserved filters

A standard eDNA slurry was prepared ensuring that the target DNA was present but diluted to an abundance closer to typical eDNA samples. The slurry was created by mixing a 4 mm diameter dorsal tail fin punch taken from an American bullfrog (*Rana (Lithobates) catesbeiana*) tadpole, a 4 mm diameter flake of Spirulina with 1 mL DNase-free TE buffer pH 8. The mixture was homogenized for 6 min at 24 Hz in a Retsch MM301 mixer mill (ThermoFisher Scientific Inc.) in microtubes containing a 3 mm tungsten carbide bead (the mixer mill rack was rotated 180° halfway through homogenization) to create a standard slurry (Additional file 1: Figure S1).

Since the purpose of this experiment was to monitor DNA integrity on filters over time measured by C_t_ values, it was crucial for all initial time points to have a positive detection. To determine the lowest concentration of DNA that resulted in 100% positive detection in all technical replicates, a tenfold serial dilution test using recirculated fresh water from the Aquatics Facility was carried out and the appropriate slurry dilution (10^–6^) was selected to create a 2 L 10× working stock (Additional file 1). Three hundred millilitre working stock was added to each of five replicates of 2700 mL recirculated water and each replicate was further divided into 1 L aliquots that were individually vacuum filtered (Additional file 1). The result was three filters with identical filtride which were distributed between each storage temperature (18, 4, and − 20 °C; Additional file 1). Each 1 L experimental sample was matched by a 1 L negative control sample of bottled distilled water (Equate brand, Walmart) for a total sample number of 30. In a laminar flow hood, all filters were quartered using forceps. Each quarter filter was stored in a separate coin envelope to allow for consistent preservation conditions and preserved using silica gel beads as described above.

#### DNA isolation and analysis

The DNA from one quarter of each filter was isolated using the DNeasy Blood and Tissue kit (QIAGEN Inc., Mississauga, ON, Canada; Cat# 69506) following the procedure outlined in [13].

The test for relatively abundant chloroplast signal using the IntegritE-DNA™ assay as described previously [13, 14] and the tests for low abundance bullfrog DNA (eFrog3 and eLICA1) were previously validated and described [13]. An additional validation step for these two assays was performed using gBlocks^®^ synthetic DNA ordered from Integrated DNA Technologies (Coralville, Iowa, United States) following the method outlined previously [14]. This allowed creation of a standard curve relating qPCR cycle threshold values to starting copy numbers, and objective, standardizable comparison of assay results. All qPCR tests followed the same run conditions outlined in [13] except that the eFrog3 assay 30 s annealing step was adjusted to 60 °C. All distilled water filter controls, positive plate controls, and no template plate negative controls performed as expected.

#### Statistical analyses

The qPCR data was analyzed with R Studio© version 1.2.1335 (2009–2019 R Studio, Inc). Data are expressed as median values to reduce the influence of outlier measurements and plotted with median absolute deviation error. Median C_t_ values for each set of eight technical qPCR replicates representing the DNA from one quarter of a filter were transformed to copies per reaction using the formula derived from each assay’s synthetic DNA standard curve. The raw data are in Additional file 2 and graphed in Additional file 3. The median copy-per-reaction values were assessed for normality using the Shapiro–Wilk test and homogeneity of variance using Levene’s test. After determining that requirements for normality and homogeneity were not met, non-parametric analyses were carried out. The Friedman repeated measures test was used to determine whether test groups contained significant differences (p ≤ 0.05), and the Wilcoxon Signed Rank test was used to determine pairwise significance between treatments within each group (p ≤ 0.06).

### Results and discussion

#### Experiment 1

**Fig. 2:**
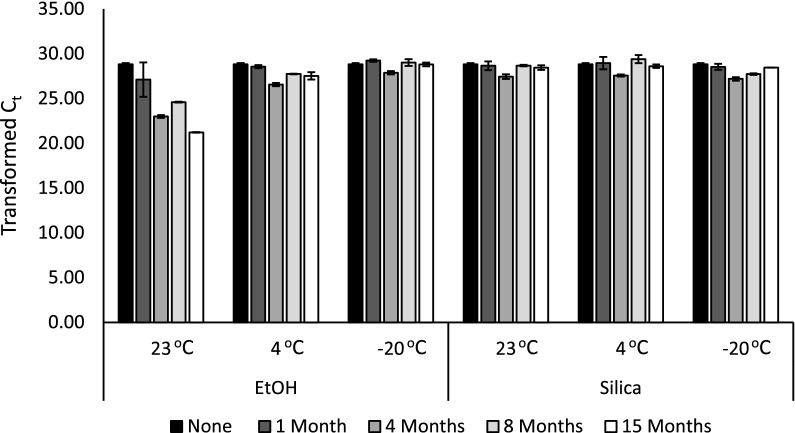
Relatively abundant eDNA is better preserved on filters desiccated with silica rather than immersed in ethanol (EtOH) at warmer temperature. Transformed cycle threshold (“Transformed C_t_”) values of IntegritE-DNA™ qPCR tests targeting chloroplast DNA isolated from filters preserved by EtOH submersion and silica desiccation (n = 2–3) at the indicated temperatures and time periods. C_t_ values were transformed by subtracting the value from 50.001 for more intuitive visualization of the change in DNA quality over time. A clear decrease in DNA quality is observed in the 23 °C EtOH-stored filters. The data are plotted as medians with median absolute deviations (error bars)

We evaluated eDNA samples for relatively abundant, naturally occurring chloroplast DNA found in water samples obtained from an outdoor tank. As expected, the filters that were immediately processed (“None” Fig. 2) returned C_t_ values between 20 and 23 (corresponding to ~ 50,000 copies/reaction) for intact samples consistent with previous observations with field water samples [14, 15]. The raw data are in Additional file 4. Filters that were stored in ethanol at 23 °C experienced a progressive decrease in eDNA sample quality (as demonstrated by the shift in C_t_ value) across all time points compared to filter samples that were processed immediately. This sample degradation was not seen in the filters that were stored with silica gel beads (Fig. 2). The detection rates of DNA isolated from the 23 °C silica gel bead preserved filters and the filters stored at − 20 or 4 °C in either ethanol or silica gel beads at any time point up to 15 months were stable as indicated by the consistent C_t_ values (Fig. 2). Majaneva et al. [9] compared ethanol to silica gel bead preservation methods for filters destined for metabarcoding analyses. They only analyzed one storage condition and time point (room temperature for 1 week) and found that silica gel desiccation yielded more consistent community composition than ethanol despite the ethanol samples having a higher concentration of DNA extracted from the filter [9]. Ethanol and silica beads performed comparably for relatively abundant target DNA over the short-term at cooler temperatures, but sample quality deteriorated in ethanol at 23 °C when stored greater than 1 month. At this temperature, we also observed that some ethanol-preserved filters began to physically degrade after 1 month of storage, and the ethanol in several samples evaporated, severely compromising sample quality.

#### Experiment 2

**Fig. 3:**
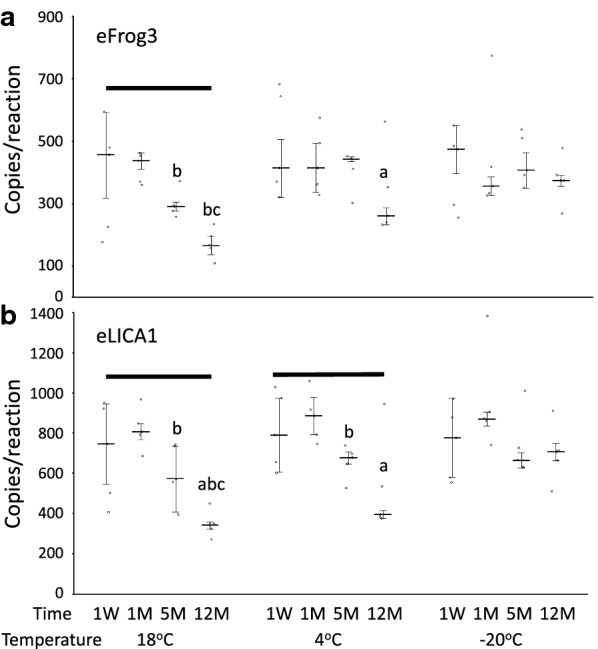
While low abundance eDNA is stable for a month when stored on silica-desiccated filters at all temperatures, storage up to a year is most stable at − 20 °C. A “standard” slurry was prepared to emulate a typical eDNA water sample which was filtered and the filters subsequently stored under silica desiccation at the indicated temperatures for 1 week (1 W), 1 month (1 M), 5 months (5 M) and 12 months (12 M) (n = 5 per temperature and time point; see Additional file 1: Figure S1 for details). The data are plotted as median starting copies per reaction (black line) for the **A** eFrog3 and **B** eLICA1 assays with median absolute deviations (error bar). Individual data points for each filter replicate are shown as open circles. Significant pairwise comparisons with 1 week, 1 month, and 5 months (Wilcoxon p = 0.06) are denoted by lowercase a, b and c, respectively. A black bar above a temperature group represents significance at p = 0.05 (Friedman) within the group

Across the three different temperature conditions, the eFrog3 test detected significant loss in DNA copy numbers over 12 months at 18 °C and the eLICA1 measured significant loss at both 18 °C and 4 °C (Friedman; p = 0.05; Fig. 3). Low abundance eDNA was preserved equally well up to 1 month regardless of storage temperature. However, a noticeable decrease in detectability was observed at 5 and 12 months when filters were stored at 18 and 4 °C (Wilcoxon; p = 0.06). Sample integrity was maintained to 12 months when filters were stored at − 20 °C (Fig. 3). The raw data are in Additional file 5.

## Conclusions

Silica gel beads are an excellent alternative to ethanol for preservation of eDNA filter samples. While short-term storage in the dark up to one month can be performed at warmer temperatures, longer-term storage should be at − 20 °C to maintain sample quality prior to DNA isolation.

## Limitations

We used three separate targeted qPCR assays and evaluated relatively abundant and low abundance DNA targets to assess the effectiveness of silica gel bead desiccation of eDNA immobilized on filters for eDNA analysis. The assessment of additional targets from a wider variety of water samples is desired.

## Supplementary Information

**Additional file 1. **Schematic of the method for creating a standard DNA slurry. Four millimeter-diameter biopsies of American Bullfrog (LICA) tail fin and a Spirulina flake were added to 1 mL TE buffer pH 8, then homogenized into a slurry. A 10^−6^ dilution using recirculated fresh water from the Aquatics Facility was made which was further diluted tenfold to the final working slurry. This dilution was determined to be optimal as it was the most dilute slurry to still obtain 100% detections for the target species. One liter final working slurry was filtered and the filter was stored at the indicated temperatures (n = 5 per temperature). Each 1 L experimental sample was matched by a 1 L negative control sample of bottled distilled water. A quarter of each filter was processed at each of 4 times (1–4) at 1 week, 1 month, 5 months, and 12 months.

**Additional file 2.** Raw data for gBlocks synthetic DNA eFrog3 and eLICA1 standard curves.

**Additional file 3.** Standard curves of gBlocks synthetic DNA serial dilution curves for (A) eFrog3 and (B) eLICA1 eDNA assays used to calculate copy number in Fig. 3. There is a very strong linear relationship between cycle threshold (C_t_) and copies/reaction.

**Additional file 4.** Raw data for Experiment 1: Comparison between ethanol and silica filter preservation of relatively abundant eDNA at three holding temperatures over time.

**Additional file 5.** Raw data for Experiment 2: Effect of holding temperature and time on low abundance eDNA on silica-preserved filters.

## Data Availability

The datasets supporting the conclusions of this article are included within the article and its additional files.

## Abbreviations

eDNA: Environmental deoxyribonucleic acid
EtOH: Ethanol
qPCR: Quantitative real time polymerase chain reaction

## References

[1] Taberlet P, Coissac E, Hajibabaei M, Rieseberg LH (2012). Environmental DNA. Mol Ecol.

[2] Deiner K, Bik HM, Machler E, Seymour M, Lacoursiere-Roussel A, Altermatt F (2017). Environmental DNA metabarcoding: transforming how we survey animal and plant communities. Mol Ecol.

[3] Goldberg CS, Turner CR, Deiner K, Klymus KE, Thomsen PF, Murphy MA (2016). Critical considerations for the application of environmental DNA methods to detect aquatic species. Methods Ecol Evol.

[4] Barnes MA, Turner CR (2016). The ecology of environmental DNA and implications for conservation genetics. Conserv Genet.

[5] Coble AA, Flinders CA, Homyack JA, Penaluna BE, Cronn RC, Weitemier K (2019). eDNA as a tool for identifying freshwater species in sustainable forestry: a critical review and potential future applications. Sci Total Environ.

[6] Goldberg CS, Pilliod DS, Arkle RS, Waits LP (2011). Molecular detection of vertebrates in stream water: a demonstration using rocky mountain tailed frogs and Idaho Giant Salamanders. PLoS ONE.

[7] Spens J, Evans AR, Halfmaerten D, Knudsen SW, Sengupta ME, Mak SST (2016). Comparison of capture and storage methods for aqueous macrobial eDNA using an optimized extraction protocol: advantage of enclosed filter. Methods Ecol Evol.

[8] Hobbs J, Goldberg CS, Helbing CC, Veldhoen N. Environmental DNA protocol for freshwater aquatic ecosystems Version 2.2. Government Publication. 2017. 48 pp. https://www.hemmera.com/wp-content/uploads/2018/08/171115-eDNA-protocol-V2.2.pdf. Accessed 11 Mar 2021.

[9] Majaneva M, Diserud OH, Eagle SHC, Bostrom E, Hajibabaei M, Ekrem T (2018). Environmental DNA filtration techniques affect recovered biodiversity. Sci Rep.

[10] Carim KJ, McKelvey KS, Young MK, Wilcox TM, Schwartz MK. A protocol for collecting environmental DNA samples from streams. Fort Collins, CO, US: Department of Agriculture, Forest Service, Rocky Mountain Research Station; 2016. Report No.: RMRS-GTR-355. 18 pp. https://www.fs.fed.us/rm/pubs/rmrs_gtr355.pdf. Accessed 11 Mar 2021.

[11] Liang Z, Keeley A (2013). Filtration recovery of extracellular DNA from environmental water samples. Environ Sci Technol.

[12] Renshaw MA, Olds BP, Jerde CL, McVeigh MM, Lodge DM (2015). The room temperature preservation of filtered environmental DNA samples and assimilation into a phenol-chloroform-isoamyl alcohol DNA extraction. Mol Ecol Resour.

[13] Veldhoen N, Hobbs J, Ikonomou G, Hii M, Lesperance M, Helbing CC (2016). Implementation of novel design features for qPCR-based eDNA assessment. PLoS ONE.

[14] Hobbs J, Round JM, Allison MJ, Helbing CC (2019). Expansion of the known distribution of the coastal tailed frog, *Ascaphus**truei*, in British Columbia, Canada using robust eDNA detection methods. PLoS ONE.

[15] Hobbs J, Adams IT, Round JM, Goldberg CS, Allison MJ, Bergman LC (2020). Revising the range of Rocky Mountain tailed frog, *Ascaphus montanus*, in British Columbia, Canada, using environmental DNA methods. Environ DNA.

