# Supplementary figures and images for: The effect of silica desiccation under different storage conditions on filter-immobilized environmental DNA
Source: BMC Res Notes. 2021 Mar 21;14:106. doi: 10.1186/s13104-021-05530-x (PMC7981917; doi:10.1186/s13104-021-05530-x)

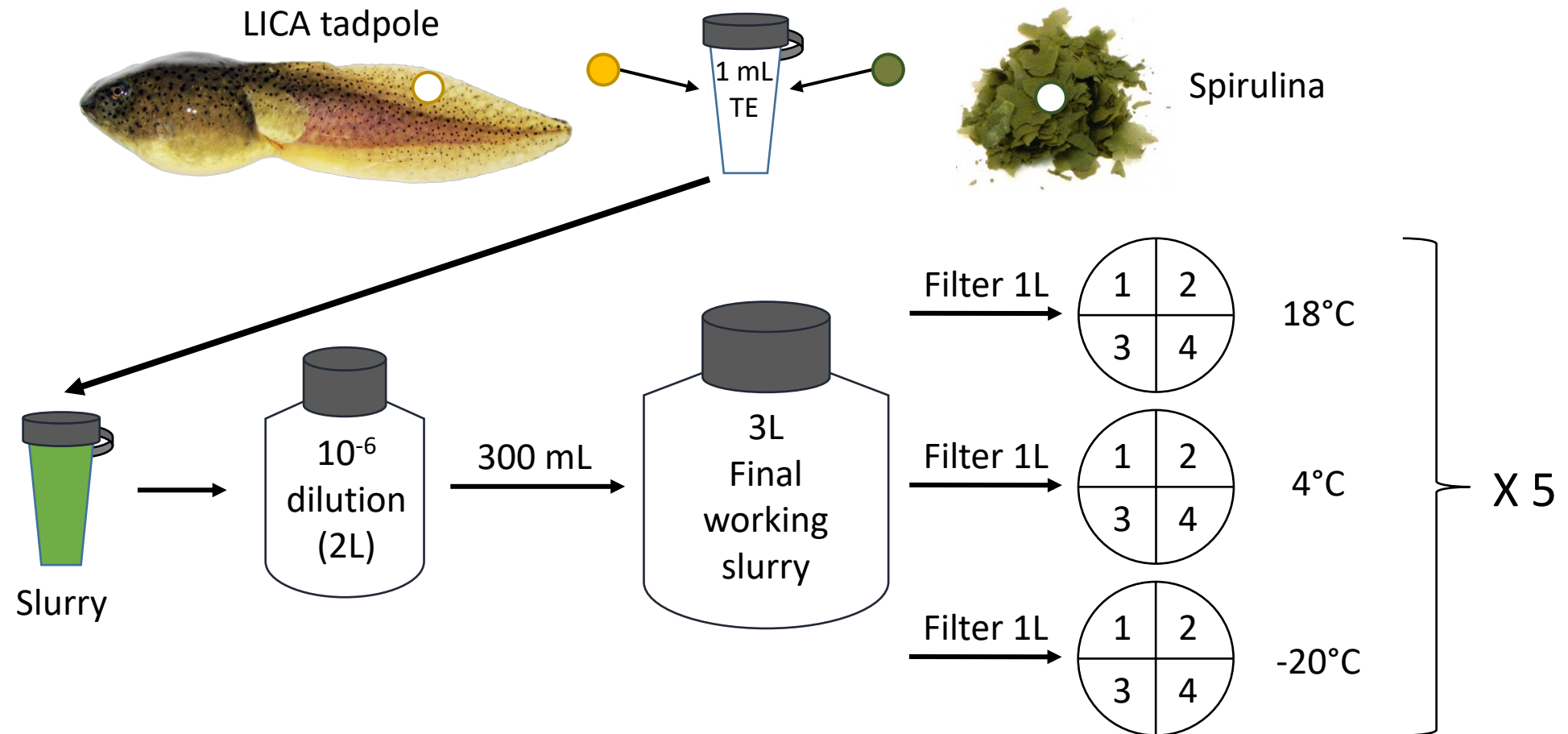

Supplement: Supplementary file 1 — Additional file 1. Schematic of the method for creating a standard DNA slurry. Four millimeter-diameter biopsies of American Bullfrog (LICA) tail fin and a Spirulina flake were added to 1 mL TE buffer pH 8, then homogenized into a slurry. A 10−6 dilution using recirculated fresh water from the Aquatics Facility was made which was further diluted tenfold to the final working slurry. This dilution was determined to be optimal as it was the most dilute slurry to still obtain 100% detections for the target species. One liter final working slurry was filtered and the filter was stored at the indicated temperatures (n = 5 per temperature). Each 1 L experimental sample was matched by a 1 L negative control sample of bottled distilled water. A quarter of each filter was processed at each of 4 times (1–4) at 1 week, 1 month, 5 months, and 12 months. [file 13104_2021_5530_MOESM1_ESM.pdf]

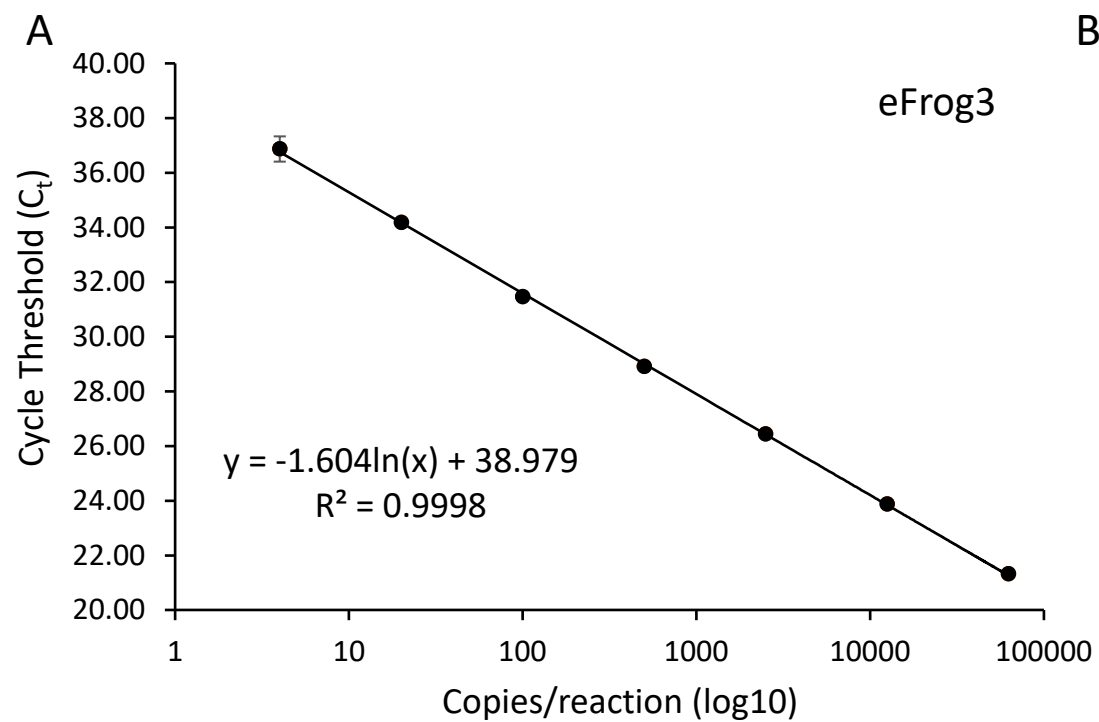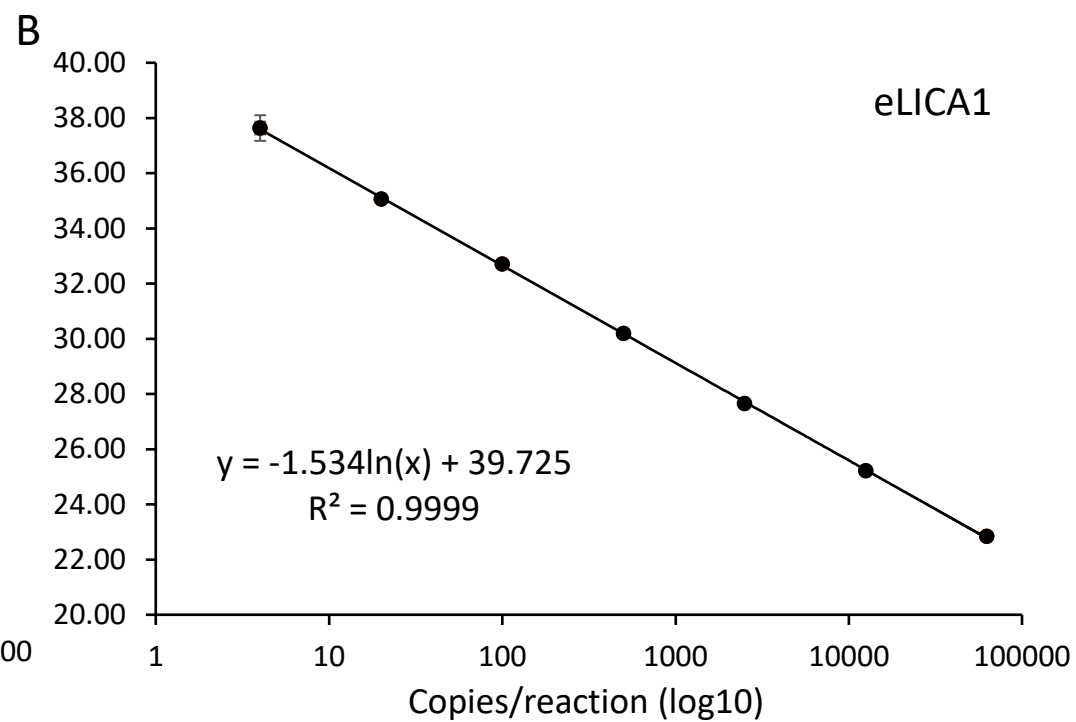

Supplement: Supplementary file 3 — Additional file 3. Standard curves of gBlocks synthetic DNA serial dilution curves for (A) eFrog3 and (B) eLICA1 eDNA assays used to calculate copy number in Fig. 3. There is a very strong linear relationship between cycle threshold (Ct) and copies/reaction. [file 13104_2021_5530_MOESM3_ESM.pdf]
